# Epigenetic Mechanisms in Osteoporosis: Exploring the Power of m^6^A RNA Modification

**DOI:** 10.1111/jcmm.70344

**Published:** 2025-01-08

**Authors:** Shuo Tian, Yagang Song, Lin Guo, Hui Zhao, Ming Bai, Mingsan Miao

**Affiliations:** ^1^ Academy of Traditional Chinese Medicine Henan University of Chinese Medicine Zhengzhou China; ^2^ Collaborative Innovation Center of Research and Development on the Whole Industry Chain of Yu‐Yao Zhengzhou China; ^3^ School of Pharmacy Henan University of Chinese Medicine Zhengzhou China

**Keywords:** BMSCs, N6‐methyladenosine, osteoblast, osteoclast, osteoporosis

## Abstract

Osteoporosis, recognised as a metabolic disorder, has emerged as a significant burden on global health. Although available treatments have made considerable advancements, they remain inadequately addressed. In recent years, the role of epigenetic mechanisms in skeletal disorders has garnered substantial attention, particularly concerning m^6^A RNA modification. m^6^A is the most prevalent dynamic and reversible modification in eukaryotes, mediating various metabolic processes of mRNAs, including splicing, structural conversion, translation, translocation and degradation and serves as a crucial component of epigenetic modification. Research has increasingly validated that m^6^A plays a vital role in the proliferation, differentiation, migration, invasion,and repair of bone marrow mesenchymal stem cells (BMSCs), osteoblasts and osteoclasts, all of which impact the whole process of osteoporosis pathogenesis. Continuous efforts have been made to target m^6^A regulators and natural products derived from traditional medicine, which exhibit multiple biological activities such as anti‐inflammatory and anticancer effects, have emerged as a valuable resources for m^6^A drug discovery. This paper elaborates on m^6^A methylation and its regulatory role in osteoporosis, emphasising its implications for diagnosis and treatment, thereby providing theoretical references.

## 1 Introduction

Osteoporosis is a condition characterised by progressive bone loss, increased bone brittleness and a heightened risk of fractures. With the acceleration of global ageing, the prevalence of osteoporosis continues to rise worldwide [1, 2]. Current treatments include bisphosphonates, selective oestrogen receptor modulators (SERMs), calcitonin and analogues of parathyroid hormone (PTH), which have been shown to mitigate bone loss and reduce fracture risk. However, these treatments are associated with several side effects, for instance, osteonecrosis of the jaw [3, 4, 5, 6], atypical femoral fractures [7, 8, 9, 10], increased risk of reproductive system tumors [11] and coronary heart disease [12], despite existing recommendations for the deprescribing of bisphosphonates in osteoporosis management [13]. Consequently, there is a pressing need to identify new therapeutic targets for osteoporosis.

Epigenetics refers to heritable modifications of gene expression that occur without changes to the nucleotide sequence [14, 15]. These epigenetic modifications primarily include DNA methylation [16, 17, 18], histone modifications [19, 20, 21] and RNA modifications [22, 23, 24], which regulate cell growth, development and differentiation [25], thereby affecting gene transcription and protein translation [26]. It is increasingly recognised that several epigenetic mechanisms are involved in osteoporosis [27], with RNA modifications playing a crucial role in maintaining bone balance, as they are regulated by the bone formation and resorption [28]. The N6‐methyladenosine modification (m^6^A) is the most prevalent mRNA base modification at the N6 position of adenosine [29, 30], which influences the metabolic processes at various stages of RNA splicing, translocation, degradation and translation [31]. m^6^A plays a key role in regulating cell differentiation and tissue development [32, 33, 34, 35]. Furthermore, m^6^A‐related regulators are implicated in the differentiation, proliferation and apoptosis of osteoblasts, osteoclasts and bone marrow mesenchymal stem cells (BMSCs), making them potential novel targets for therapeutic interventions in osteoporosis [36].

Inflammation disrupts the balance between bone destruction and bone formation, accelerating osteoporosis and serving as a risk factor for its development [37, 38]. Compounds derived from traditional medicines with anti‐inflammatory properties may mitigate the progression of osteoporosis [39]. Cumulative evidence suggests that several well‐known anti‐inflammatory natural products, including polyphenols (e.g. curcumin [40], resveratrol [41]), flavonoids (e.g. quercetin [42], baicalin [43]) and alkaloids (e.g. betaine [44], clause E [45, 46]), exhibit potential m^6^A‐targeting regulatory effects in inflammatory diseases. At present, the development of m^6^A‐targeting drugs is progressing through three distinct stages: the utilisation of traditional medicine‐based natural products, modern chemical modification or synthesis and the application of artificial intelligence (AI)‐assisted methodologies for future advancements [47]. Notably, the efficacy and safety profiles of natural products and their derivatives from traditional medicines present promising potential for m^6^A‐targeted therapeutic interventions. Therefore, targeting m^6^A regulators may represent a prospective therapeutic strategy for osteoporosis.

Notably, m^6^A presents new potential targets for anti‐osteoporosis interventions, carrying significant implications for the prevention, diagnosis and treatment of osteoporosis. This article reviews the current research status of m^6^A in relation to osteoporosis and provides a foundational reference for precise and personalised treatment approaches.

## 2 Regulators of m^6^A Methylation

**FIGURE 1:**
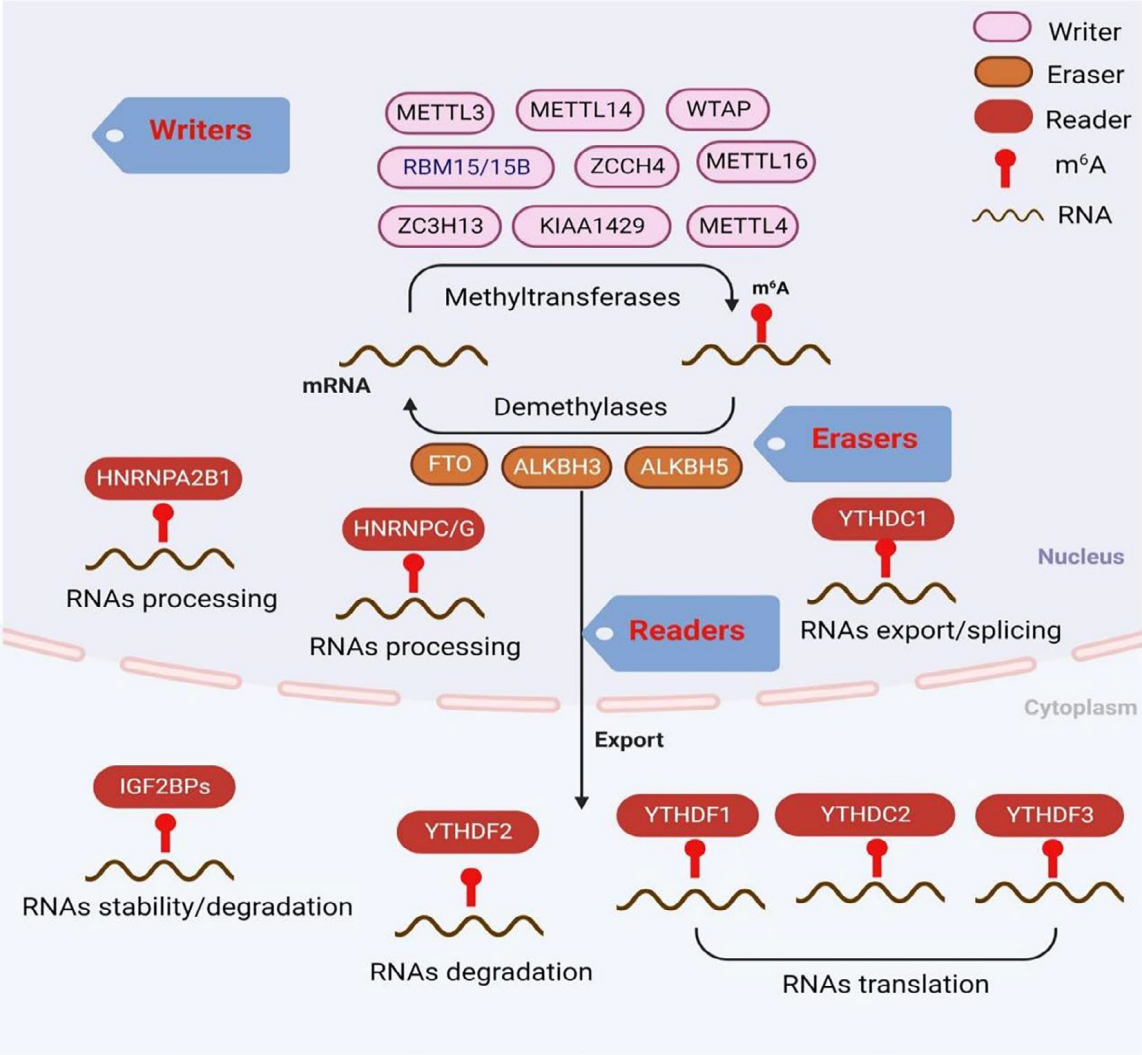
Mechanism of m^6^A modification. The process of m^6^A is installed by ‘writers’, ‘erasers’ and ‘readers’.

As the central dogma of molecular biology, genetic information flows from DNA through RNA to proteins [48, 49]. RNA serves as a fundamental component in genetic regulation mechanisms [50] and plays a crucial role in regulating complex biological processes at various levels [51]. RNA modification represents an important regulatory pathway in post‐transcriptional RNA processes [52, 53], with over 150 distinct RNA modifications identified [54], including N1‐methyladenosine (m^1^A) [55], 5‐methylcytidine (m^5^C) [56, 57], m^6^A [58], 7‐methylguanosine (m^7^G) [59, 60], ribose methylations (2′‐O‐Me) [61, 62] and pseudouridine (Ψ) [63, 64]. These modifications are prevalent across various types of RNA, such as transfer RNA (tRNA), messenger RNA (mRNA), ribosomal RNA (rRNA), long non‐coding RNA (lncRNA) and small non‐coding RNA (sncRNA). They significantly influence the structure and function of tRNA and rRNA, the efficiency and stability of mRNA translation, microRNA processing, cellular differentiation and the internal perceptual pathway of pathogens [65, 66]. Analogous to DNA methylation and histone methylation, the m^6^A modification is also dynamic and reversible [67, 68], primarily mediated by ‘writers’ (methyltransferases), ‘erasers’ (demethylases) and ‘readers’ (binding proteins) [69]. The detailed mechanism of the m^6^A process is illustrated in Figure 1.

### 2.1 Methyltransferases

m^6^A is catalysed by the methyltransferase complex (MTC), which includes essential components known as methyltransferases or ‘writers’ involved in the methylation reaction of RNAs [70]. These components encompass methyltransferase‐like 3 (METTL3) [71], methyltransferase‐like 14 (METTL14) [72], methyltransferase‐like 16 (METTL16) [73], methyltransferase‐like 4 (METTL4) [74], methyltransferase‐like 5 (METTL5) [75], Wilms tumour 1‐associated protein (WTAP) [76], KIAA1429 (VIRMA, a vir‐like m^6^A methyltransferase associated) [77, 78], RNA‐binding motif protein 15/15B (RBM15/RBM15B) [79], zinc finger CCCH‐type containing 13 (ZC3H13) [32] and zinc finger CCHC‐type containing 4 (ZCCHC4) [80]. METTL3 plays a pivotal role in the m^6^A MTC, primarily catalysing the m^6^A modification [81], METTL14 significantly contributes to stabilising METTL3 and recognising target RNAs; it acts as a pseudo‐methyltransferase [82]. Together, METTL3 and METTL14 form steady compounds in a 1:1 ratio and aggregate within nuclear speckles. Although WTAP lacks catalytic function, it aids in the localisation of METTL3 and METTL14 within nuclear patches. Other components, such as ZC3H13 and RBM15, have been shown to directly modulate m^6^A modification. METTL4, which does not possess a catalytic structure, assists METTL3 in enhancing its catalytic efficiency. KIAA1429 serves as the primary scaffolding component of the MTC, mainly regulating the 3′‐UTR and regions near the termination codon of m^6^A [83, 84]. METTL16 functions as an independent methyltransferase and plays a crucial role in regular splicing [85].

### 2.2 Demethylases

Demethylases, commonly referred to as ‘erasers’, primarily mediate the demethylation of m^6^A modification in RNA by wiping off the methyl group of m^6^A. This group mainly includes FTO and ALKB homolog 5 (ALKBH5), both of which belong to the family of alpha‐ketoglutarate‐dependent dioxygenases. FTO was the first identified ‘eraser’ discovered in 2011 [86]; it oxidises m^6^A from N6‐hydroxymethyladenosine to N6‐formyladenosine in a Fe(II)‐ and α‐KG‐dependent manner [47, 87], subsequently hydrolysing it into adenosine, thus completing the demethylation process. ALKBH5, identified as the second ‘eraser’ in 2013, functions as an FTO homolog [88] and plays a crucial role in maintaining the homeostasis of m^6^A modifications during transcription. Recent studies have also indicated that ALKB homolog 3 (ALKBH3) performs demethylation through a mechanism similar to that of FTO and ALKBH5 [89].

### 2.3 Readers

The primary role of binding proteins, commonly referred to as ‘readers’, is to recognise information regarding RNA methylation modifications and to facilitate downstream processes such as mRNA variable cleavage, out‐of‐nucleus translocation, translation, degradation and miRNA processing. Consequently, the target RNAs exhibit diverse biological functions [90]. The YT521‐B homology (YTH) domain family, the insulin‐like growth factor 2 mRNA‐binding proteins domain family (IGF2BP) and the heterogeneous nuclear ribonucleoprotein (HNRNP) family are the most prevalent ‘readers’. Members of the YTH family, including YTH domain family proteins1‐3 (YTHDF1‐3) and YTH domain‐containing proteins 1–2 (YTHDC1‐2), preferentially interact with RNA that contains m^6^A modifications. YTHDF2 was the first identified m^6^A ‘reader’ and is known to promote mRNA degradation [91]. YTHDF1 enhances the translation of target RNAs by binding to the m^6^A site located near the stop codon [92]. YTHDF3 affects both the translation and degradation of target RNAs by interacting with YTHDF1 and YTHDF2, thereby affecting their function [93]. YTHDC1 is involved in regulating the export of m^6^A‐modified RNA from the nucleus and in splicing [94, 95]. YTHDC2 enhances the efficiency of translation while reducing the abundance of target RNAs [96, 97]. The IGF2BP family, which includes IGF2BP1, IGF2BP2 and IGF2BP3, functions to enhance the expression of target mRNA by stabilising these RNAs [98]. The HNRNP family, primarily comprising HNRNPA2B1, HNRNPC and HNRNP G [99], adjusts the processing of RNA substrates [100, 101]. As research has progressed, a number of additional ‘readers’ have been identified, including proline‐rich coiled‐coil 2A (PRRC2A) [102], Hu‐Antigen R (HuR) [103, 104] and fragile X mental retardation 1 (FMR1) [105].

## 3 Roles of m^6^A Methylation in Osteoporosis

**FIGURE 2:**
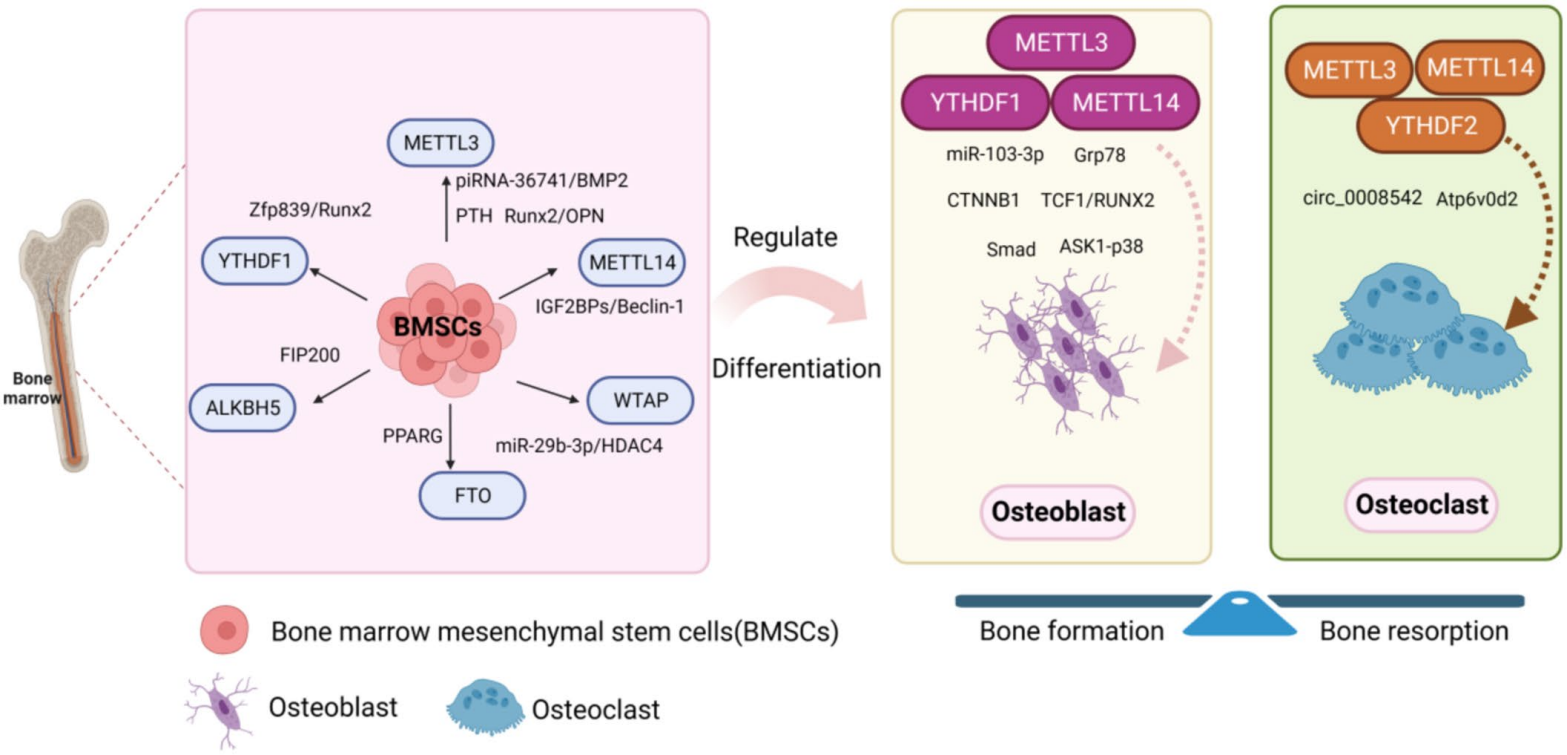
The role of m^6^A in the progression of osteoporosis. m^6^A in osteoporosis mainly affects the osteogenic differentiation of BMSCs, as well as the proliferation and differentiation of osteoblasts and the differentiation of osteoclasts.

The functions of bones require the maintenance of both bone shape and bone density, which relies on the process of bone remodelling [106]. Bone remodelling is dependent on osteoblast‐mediated bone formation and osteoclast‐mediated bone resorption [107]. In healthy adults, the balance of bone homeostasis is maintained in dynamic equilibrium, regulated by both chemical and mechanical factors. Osteoporosis occurs when this dynamic balance is disrupted [108, 109]. Recent studies have indicated that m^6^A and other regulatory factors can modulate the pathological phenotype of osteoporosis [110] and are key contributors to osteoporosis (Figure 2).

### 3.1 m^6^A Methylation and BMSCs

BMSCs are adult multipotent stem cells derived from the bone marrow cavity, with the potential for multi‐lineage differentiation and self‐renewal ability [111]. These cells can differentiate into osteoblasts, adipocytes and chondrocytes. BMSCs continuously undergo mitosis and migration and differentiate into osteoblasts under the influence of various transcription factors such as Runx2 [112], PPARγ [113], hormones [114], physicochemical factors [115] and certain biological or pathological stimuli [116]. BMSCs serve as the primary source of osteoblasts [117] and also provide a cellular basis for bone growth and repair. They play a crucial role in regulating bone metabolism, promoting bone formation and maintaining normal bone stability.

The osteogenic differentiation of BMSCs mainly undergoes the following steps. Firstly, BMSCs differentiate into osteochondral progenitor and osteochondral progenitor cells are characterised by osteoblast progenitor cells. Secondly, these osteoblast progenitor cells proliferate and develop into preosteoblast cells, which enter a phase of rapid proliferation. As proliferation progresses, the capacity for cell division gradually decreases. During this phase, genes associated with extracellular matrix (ECM) maturation, such as alkaline phosphatase, type I collagen and matrix calprotectin, become further activated. At this stage, osteoblasts synthesise and secrete an organic matrix, primarily composed of bone‐like material (mainly formed by type I collagen) and mature osteoblasts express proteins related to ECM calcification, mainly osteocalcin. This process leads to the formation and subsequent mineralisation of the bone matrix. Finally, mature osteoblasts become encapsulated within the newly formed bone matrix and undergo terminal differentiation into osteoblasts, thereby completing the osteogenesis process.

Recent evidence shows that m^6^A and its modulators can regulate the differentiation of lipogenic and osteogenic in BMSCs, highlighting their close relationship with BMSCs differentiation, The methylases involved include METTL3, METTL14, WTAP, FTO, ALKBH5 and YTHDF1. Notably, most research has primarily concentrated on METTL3, which has been shown to mediate m^6^A modifications that influence the destiny of BMSCs [118]. Specifically, METTL3 was an altitudinal expression during the osteogenic differentiation of BMSCs and silencing METTL3 significantly impairs the ability of osteogenic differentiation [119]. Herein, a brief review of studies on m^6^A in BMSCs is performed in Table 1.

**TABLE 1:** Role of m^6^A in BMSCs.

m^6^A regulators	Roles in m^6^A	Mechanism	Function in osteoporosis	References
METTL3	Writers	LncRNA MIR99AHG	Strengthen osteogenic differentiation	[120]
METTL3	Writers	Wnt signalling pathway	Promote osteogenic potential	[121]
METTL3	Writers	LINC00657/miR‐144‐3p/BMPR1B axis	Promote osteogenic differentiation	[122]
METTL3	Writers	Precursor‐miR‐320/RUNX2	Promote osteogenic differentiation	[123]
METTL3	Writers	Parathyroid hormone (PTH)/parathyroid hormone receptor‐1 (Pth1r)	Modulate osteogenesis and adipogenesis	[118]
METTL3	Writers	Runx2/OPN	Strengthen osteogenic differentiation	[124]
METTL3	Writers	piRNA‐36741/BMP2	Promote osteogenic differentiation	[125]
METTL3	Writers	vegfa‐164 and vegfa‐188	Modulate osteogenic differentiation	[119]
METTL3	Writers	HIF‐1α, PI3K/Akt and Hippo	Strengthen osteoclast generation	[126]
METTL3	Writers	Glycolytic pathway	Modulate osteogenesis	[127]
METTL3	Writers	IGF2BP1/m^6^A/RUNX2	Strengthen osteogenic differentiation	[128]
METTL3	Writers	circCTTN	Promote osteogenic differentiation of hUCMSCs	[129]
METTL14	Writers	SMAD1	Strengthen osteogenic differentiation	[130]
METTL14	Writers	IGF2BPs/Beclin‐1	Strengthen osteogenic differentiation of BSMCs	[131]
METTL14	Writers	pri‐miR‐873	Strengthen osteogenic proliferation and differentiation	[132]
METTL14	Writers	PTPN6	Modulate osteogenic differentiation	[133]
METTL14	Writers	P4HB	Promote osteogenic differentiation	[134]
WTAP	Writers	miR‐29b‐3p/HDAC4 axis	Promote osteogenic differentiation	[135]
WTAP	Writers	miR‐181a and miR‐181c/SFRP1	Modulate the differentiation fate of BMSCs	[136]
FTO	Erasers	Runx2 mRNA	Restraint osteogenic differentiation	[137]
FTO	Erasers	FTO‐PPARG axis	Promote osteogenic differentiation of BMSCs	[138]
FTO	Erasers	GDF11‐FTO‐PPARγ axis	Inhibit bone formation	[139]
ALKBH5	Erasers	FIP200	Attenuate apoptosis of nucleus pulposus cells	[140]
YTHDF1	Readers	Zfp839/Runx2	Promote osteogenesis of BMSCs	[141]
YTHDF2	Readers	FBLN1/miR‐615‐3p	Restraint osteogenic differentiation of WJCMSCs	[142]

### 3.2 m^6^A Methylation and Osteoblast

Osteoblasts are essential for bone remodelling and are derived from BMSCs [143]. Their vitality is determined by the proliferation and differentiation of osteoblasts, as well as the presence of mature osteoblasts. The processes of osteoblast proliferation and differentiation are regulated by multiple nuclear proteins [144], including runt‐related transcription factor 1 (Runx1), Indian hedgehog (Ihh), runt‐related transcription factor 2 (Runx2), osterix, activator protein 1 (AP1) and activating transcription factor 4 (ATF4) [145]. However, the regulatory mechanism governing osteoblast function remains largely unknown. Emerging studies have shown that m^6^A plays a vital role in regulating osteoblast proliferation and differentiation, with current research focusing on METTL3, METTL14 and YTHDF1. There are diverse reports regarding the functions of METTL3 in osteoblasts. One study indicates that in MC3T3‐E1 cells, down‐regulation of METTL3 by mediation of the miR‐7212‐5p maturation can promote osteogenic processes [146]. Conversely, another study shows that the knockdown of METTL3 inhibits osteoblast differentiation through the stabilising of Smad7 and Smurf1 in the context of LPS‐induced inflammation [147]. These results may be due to the different states of physiological and pathological states and the expression of m^6^A varies. A review of research on m^6^A in osteoblasts is presented in Table 2.

**TABLE 2:** Role of m^6^A in osteoblasts.

m^6^A regulators	Roles in m^6^A	Mechanism	Function in osteoporosis	References
METTL3	Writers	METTL3/ASK1‐p38 signalling pathway	Inhibit ferroptosis of osteoblasts	[148]
METTL3	Writers	Smad signalling	Inhibit osteoblast differentiation	[147]
METTL3	Writers	Enhanced Grp78 expression	Promote osteoblast apoptosis and inhibit cell proliferation and differentiation	[149]
METTL3	Writers	CTNNB1	Inhibit the stemness remodelling of prostate cancer (PCa) cells by osteoblasts	[136]
METTL3	Writers	miR‐7212‐5p/FGFR3 axis	Modulate osteogenic processes	[146]
METTL3	Writers	Igf2bp2‐Slc1a5 axis	Promote osteoblast senescence	[150]
METTL3	Writers	Hspa1a stability	Inhibit osteoblast ageing	[151]
METTL3	Writers	SOX4	Modulate the proliferation and differentiation of osteoblasts	[152]
METTL14	Writers	TCF1/RUNX2 axis	Increase osteogenic activity	[153]
METTL14	Writers	miR‐103‐3p/METTL14/m6 A signalling axis	Inhibit osteoblast activity	[154]
FTO	Erasers	PDIA3/FTO/USP20	Modulate osteogenic differentiation	[155]
YTHDF1	Readers	YTHDF1/THBS1 pathway	Modulate osteogenic differentiation	[156]

### 3.3 m^6^A Methylation and Osteoclast

Osteoclasts play a crucial role in bone remodelling and maintaining skeletal integrity, differentiating from precursor cells of monocyte or macrophage lineage [157] and undergoing proliferation, differentiation, fusion and activation of precursor cells. Excessive osteoclast activity can lead to bone loss, contributing to various bone diseases [158]. Cytokines and their receptors regulate the process of osteoclastogenesis, with multiple soluble factors and transcription factors identified that influence osteoclast proliferation and differentiation. Notable among these are macrophage colony‐stimulating factor (M‐CSF), receptor activator of NF‐κB ligand (RANKL), cellular oncogene fos (c‐Fos), nuclear factor of activated T cells 1 (NFATc1) and nuclear factor kappa beta (NF‐κB) [159]. The role of m^6^A in osteoclasts has been the focus of several studies; for instance, the expression of METTL3 increases during osteoclast differentiation through the expression and stability of Atp6v0d2 via YTHDF2 [160]. Inhibiting METTL3 can reverse bone resorption and osteoclastogenesis by promoting the interaction between circ_0008542 and miRNA‐185‐5p [161]. METTL14 inhibits bone resorption and osteoclast differentiation via the GPX4‐m^6^A‐HuR axis [162], with METTL14 overexpression not only inhibiting osteoclast differentiation but also promoting osteoblast differentiation by the harmonisation of SIRT1 mRNA m^6^A [163]. Overexpression of ALKBH5 mitigates the circ_0008542‐induced bone loss by disrupting the combining between circ_0008542 and the miR‐185‐5p/RANK axis [161]. YTHDC1 enhances PTPN6 RNA stability and inhibits osteoclast differentiation in an m^6^A‐HUR‐dependent manner [164]. Furthermore, depletion of YTHDF1 reduces the phosphorylation levels of key proteins in the NF‐κB, MAPK and PI3K‐AKT pathways, thereby affecting the stability of TNFRSF11a mRNA, a critical molecule in the activation of these upstream signalling pathways, ultimately inhibiting osteoclastogenesis [165].

### 3.4 Transcription Factors

Transcription factors are protein molecules characterised by a unique structure that regulates gene expression. They can be categorised into universal and specific transcription factors based on their functional characteristics. Currently, several specific transcription factors including runt‐related transcription factor 2 (Runx2), β‐catenin, Osterix (Osx), activator protein‐1 (AP‐1) and activating transcription factor 4 (ATF4) have been identified as influencing the differentiation of BMSC_S_ or osteoblasts through m^6^A modification.

Runx2, also known as core‐binding factor alpha 1 (CBFA1), polyomavirus enhancer‐binding protein 2αA (pEBp2αA) and acute myeloid leukaemia factor 3 (AML3), is a member of the runt structural domain gene family. It serves as an osteogenic differentiation‐specific transcription factor that regulates the transcription of numerous genes, which is both necessary and sufficient for the differentiation of mesenchymal stromal cells into the osteoblast lineage. Studies suggest that Runx2 triggers the formation of bone matrix proteins during the early stages of osteogenic differentiation while simultaneously maintaining osteoblasts at an earlier stage and preventing their further differentiation, resulting in a substantial number of immature osteoblasts [166, 167]. Molecular mechanism studies have shown that Runx2 mRNA is an m^6^A‐methylated target of METTL3 at its 3′‐UTR [128], METTL3 mediates m^6^A methylation of Runx2, enhancing cellular stability and potentially rescuing the characteristics of osteoporosis [123]. Additionally, FTO can directly bind to Runx2, reducing both the m^6^A methylation level and the overall mRNA expression of Runx2 [168], thereby inhibiting osteogenic differentiation and promoting osteoporosis [137]. YTHDC2 has been shown to accelerate RUNX2 mRNA degradation through m^6^A methylation, inhibiting the osteogenic differentiation of rat BMSCs [169]. Meanwhile, IGF2BP1 can enhance the stability of RUNX2 mRNA [170] and ALKBH5 can extend the half‐life of Runx2 transcripts, influencing osteogenic differentiation [171].

Osterix, also known as SP7, is a transcription factor characterised by a zinc finger structure that is specifically expressed by osteoblasts and is restricted to developing bone tissue and it plays a crucial role in the directional differentiation of preosteoblasts to immature osteoblasts. Osterix can be activated by its binding to Runx2 binding elements and functions downstream of Runx2 [172]. Studies have demonstrated that the knockdown of Mettl3 in BMSCs leads to a reduction in Osterix mRNA expression; however, studies concerning the level of m^6^A modification of Osx remain limited.

Several bone formation markers, such as alkaline phosphatase (ALP) and osteocalcin (OCN), play crucial roles in bone metabolism. ALP is produced during the early stages of osteoblast mineralisation and exhibits a positive correlation with the rate of bone formation. Conversely, OCN is considered a marker of late bone formation. Recent studies have indicated that m^6^A modification affects the proliferation, differentiation and apoptosis of bone‐related cells, such as BMSCs, osteoblasts and osteoclasts, by regulating the mRNA expression of ALP, OCN and other associated genes. However, there has yet to be any study into the changes in m^6^A methylation modification of ALP and OCN.

### 3.5 Signalling Pathways

The development and regeneration of skeletal tissue is a complex, multistep and highly regulated process involving multiple signalling pathways. Among these, the SMAD1/5/8, PI3K/AKT and Wnt/β‐catenin signalling pathways play crucial roles in the regulation of osteogenic differentiation, particularly in osteoblast differentiation.

#### 3.5.1 BMP‐Smad Signalling Pathway

Bone morphogenetic protein (BMP) is an important growth factor in bone tissue. Upon binding to autocrine and paracrine BMP ligands, BMP activated Smad‐dependent pathways lead to the recruitment of Runx2, which in turn activates osteogenic gene expression [173, 174] and promotes osteogenic differentiation. It has been shown that METTL3 regulates the stability and half‐life of Smad7 and Smurf1 through YTHDF2 under inflammatory conditions [147]. In contrast, Smad7 can negatively regulate Smad signalling via the ubiquitin proteasomal degradation of Smad1/5/9 mediated by Smurf1 [175], thereby affecting osteogenic differentiation. Additionally, METTL14 binds to Smad1 at the 1739‐1‐1743 bp site; the knockdown of METTL14 results in increased Smad1 degradation and inhibits the m^6^A methylation modification of Smad1, thereby inhibiting the osteogenic differentiation of BSMCs [130].

#### 3.5.2 Wnt/β‐Catenin

Wnt proteins are members of a family of secreted molecules and the Wnt signalling pathway has been proposed as an alternative exogenous BMP pathway with a certain degree of osteogenic potential. When the classical pathway is activated, accumulated β‐catenin proteins translocate to the nucleus, inducing the transcription of target genes (e.g. c‐Myc) [176], which leads to the transformation of mesenchymal stromal cells into osteoblasts and contributes to the differentiation and metabolism of osteoblasts. It has been shown that the inhibition of METTL3 increases the expression of negative regulators of the Wnt signalling pathway. Under LPS‐stimulated conditions, METTL3 mediates Wnt/β‐catenin‐induced transcription of the target gene c‐Myc, which promotes osteoblast ATP production, ribosome biogenesis and osteoblast differentiation [177].

#### 3.5.3 PI3K/AKT

**FIGURE 3:**
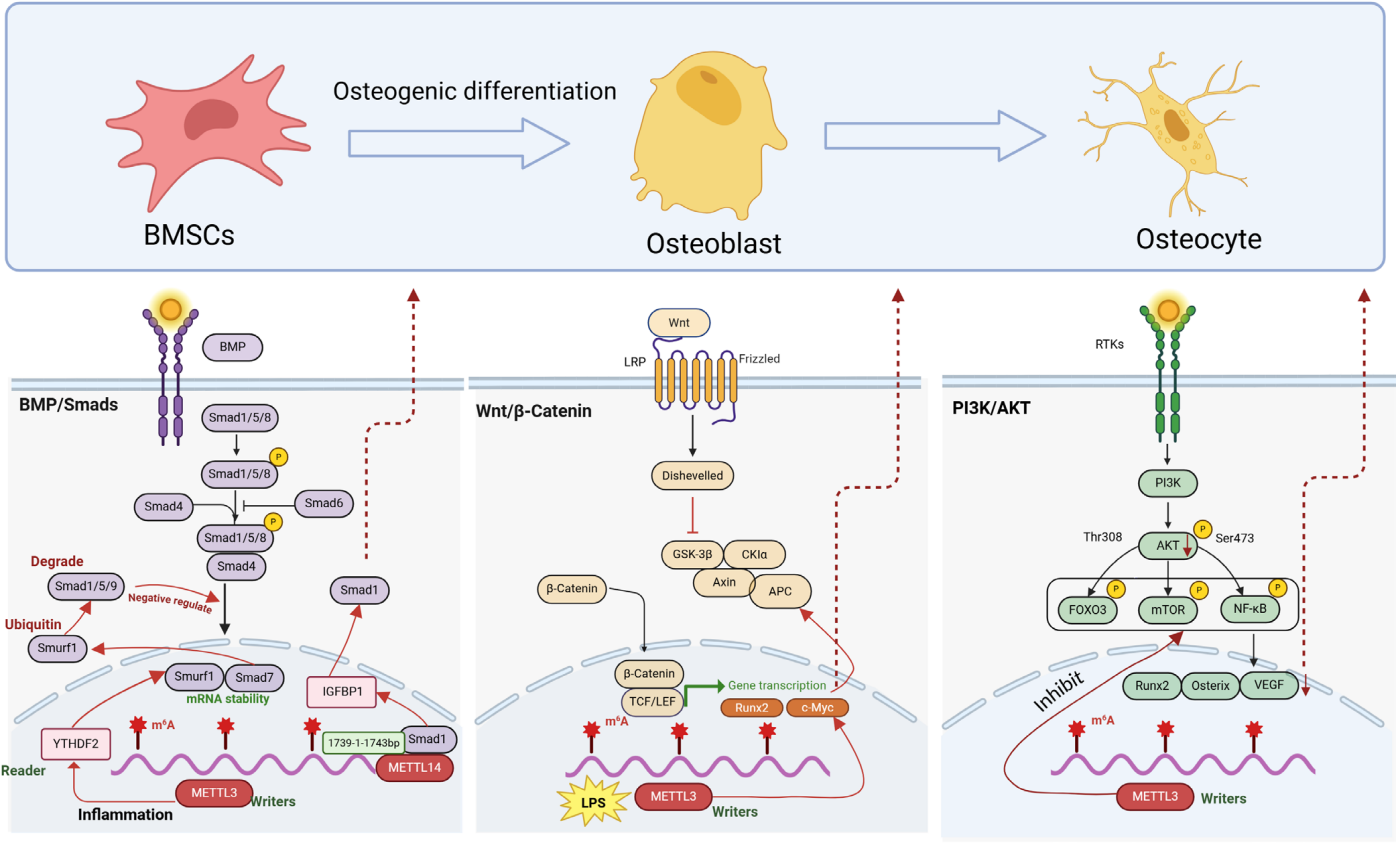
m^6^A and the regulation of major signalling pathways.

Phosphoinositide 3‐kinase (PI3K) is an enzyme that catalyses the phosphorylation of one or more inositol phospholipids at the 3‐position of the inositol ring. Type I PI3K primarily synthesises the phospholipid PIP3, which is distributed to various cell surface receptors that regulate cell motility, growth, survival and differentiation [178]. Activated PIP3 functions by recruiting serine/threonine kinase (Akt) from the cytoplasm, translocating it to the cell membrane by binding to the pleckstrin homology (PH) structural domain at the N‐terminal end of Akt. This activation is aided by 3‐phosphoinositide‐dependent Kinase‐1 (PDK1) and 3‐phosphoinositide‐dependent Kinase 2 (PDK2), which phosphorylate the threonine phosphorylation site (Thr308) and the serine phosphorylation site (Ser473) on the Akt protein, respectively. This activation subsequently influences the expression of downstream proteins, such as nuclear factor κB (NF‐κB) and forkhead box class O proteins (FOXOs), which are key regulators of bone tissue metabolism and osteogenic differentiation [179]. Furthermore, the knockdown of METTL3 in BMSCs has been shown to inhibit the PI3K‐Akt signalling pathway, resulting in significantly reduced Akt phosphorylation levels. This leads to the downregulation of bone formation‐related genes (e.g. Runx2 and Osterix) and vascular endothelial growth factor (VEGF) [119] was restricted, as well as a decrease in the translation efficiency of parathyroid hormone receptor‐1 (PTHR) mRNA [118] (Figure 3).

### 3.6 Non‐Coding RNA

Non‐coding RNAs (nc RNAs) are transcribed from the genome [180] and do not encode proteins instead, they perform their respective biological functions at the RNA level. This category mainly includes microRNAs (miRNAs), long noncoding RNAs (lncRNAs) and circular RNAs (circRNAs) [181, 182]. Studies have demonstrated that nc RNAs influence disease progression through two pathways; one stemming from their own disorders and the other involving lncRNAs and circRNAs, which can act as competing endogenous RNAs for various microRNAs in order to regulate the expression of miRNA‐targeted genes, thereby affecting different biological pathways and playing a significant role in the regulation of bone metabolism. Research has indicated that miR‐615‐3p mediates the osteogenic differentiation and bone regeneration of WJCMSCs [142] by regulating the functions of FBLN1 at the 3′UTR site via YTHDF2 through an m^6^A‐miRNA regulatory mechanism. Additionally, the overexpression of miR‐873 inhibits the proliferation and differentiation of BMSCs, primarily due to the modification of METTL14 m^6^A, which promotes the processing of pri‐miR‐873 into mature miR‐873 by binding to DGCR8 in BMSCs [132]. Furthermore, METTL14‐dependent m^6^A methylation inhibits the processing of miR‐103‐3p by DiGeorge critical region 8 (DGCR8) and promotes osteoblast activity [154]. miR‐29b‐3p has been identified as a downstream target of WTAP, which interacts with DGCR8 to enhance the maturation of pri‐miR‐29b‐3p in an m^6^A‐dependent manner via the histone deacetylase 4 (HDAC4) binding site, thereby regulating the differentiation of BMSCs [135]. Other studies have reported that WTAP promotes the osteogenic differentiation of BMSCs by inhibiting SFRP1 mRNA expression through the methylation of pri‐miR‐181a and pri‐miR‐181c, which are recognised by YTHDC1, leading to increased maturation of miR‐181a and miR‐181c [183]. Additionally, METTL3‐mediated methylation of lncRNA MIR99AHG enhances the osteogenic differentiation of BMSCs by targeting miR‐4660 [120]. Dual luciferase reporter gene assays have demonstrated that miR‐144‐3p can interact with either LINC00657 or BMPR1B, with METTL3 mediating m^6^A methylation of LINC00657 and upregulating BMPR1B expression via miR‐144‐3p to promote osteogenic differentiation of BMSCs [122]. Furthermore, piRNA‐36,741 overexpression promotes osteogenic differentiation of BMSCs through METTL3‐mediated m^6^A methylation of BMP2 transcripts [125]. Lastly, Circ_0008542 in osteoblast exosomes promotes osteoclast‐induced bone resorption through m^6^A methylation [161].

### 3.7 Others

Autophagy plays a crucial role in the regulation of bone metabolism, and increasing evidence suggests that m^6^A‐related enzymes are involved in the production of autophagy. METTL14 regulates the osteogenesis of BMSCs by inducing autophagy through the m^6^A/IGF2BPs/Beclin‐1 signalling axis [131]. The tricarboxylic acid (TCA) cycle in mitochondria is a key process for cellular energy production and redox homeostasis [184]. Recent studies have highlighted the significance of mitochondria in the pathogenesis of osteoporosis. It has been shown that TCA cycle enzymes or intermediates are essential for epigenetic pathways that can be modified by RNA demethylase enzymes, thereby altering the chromatin accessibility of target gene transcripts [185] or influencing the lineage commitment of stem cells [186]. The interplay between the TCA cycle and epigenetics in maintaining bone homeostasis warrants a detailed review. Furthermore, it has been reported that METTL3 affects the stability of ACLY and SLC25A1 mRNAs through the m^6^A reading proteins IGF2BP2 and IGF2BP2/3, which in turn influence ATP glycolysis and thus regulate the osteogenic differentiation of dental pulp stem cells [127].

## 4 Clinical Application of m^6^A in Osteoporosis

With developments in molecular biology and a comprehensive understanding of osteoporosis, molecularly targeted therapeutic drugs have ushered in a new era. The investigation of anti‐osteoporosis drugs that target various mechanisms of action represents an effective way to treat osteoporosis, which could more accurately regulate the dynamic balance between bone resorption and formation. Furthermore, as previously noted, m^6^A has significant potential as a biomarker for the diagnosis and treatment of osteoporosis.

Studies have shown that the expression levels of METTL3 and METTL14 are significantly decreased in the bone tissue of osteoporosis patients [123]. Consequently, METTL3 and METTL14 may serve as potential targets for osteoporosis treatment. Research has found that STM2457 can inhibit the catalytic activity of METTL3/METTL14, targeting populations of acute myeloid leukaemia (AML) key stem cells with an IC_50_ = 16.9 nM and it is identified as a specific METTL3 inhibitor [187]. Additionally, UZH1a, a small‐molecule METTL3 inhibitor, exhibits cellular activity and high nanomolar potency in biochemical experiments, along with good selectivity for m^6^A methylase. The binding mode of UZH1a in relation to the catalytic activity of METTL3/METTL14 has been elucidated, providing a foundation for the development of potent inhibitors [188]. High‐throughput docking was used to screen analogues and derivatives targeting METTL3, with the binding mode subsequently verified by protein crystallography. Two compounds predicted to be METTL3 inhibitors attended to good efficiency, offering a pathway for the development of METTL3 inhibitor [189]. Current research has focused on inhibitors; however, it is important to note that the upregulation of METTL3 may also contribute to the treatment of osteoporosis. Activators METTL3 could represent a promising therapeutic approach, but further investigations are ongoing.

Genome‐wide research has identified BMD‐associated m^6^A‐SNPs that play prominent effects in the onset of osteoporosis [190]. Clinical studies have demonstrated the close connection between variations in the FTO gene and hip fracture risk [191], confirming that FTO SNPs are correlated with variations in bone mineral density (BMD), potentially establishing them as new biomarkers for osteoporosis [192]. Several cell‐active FTO inhibitors have been discovered. Rhein, the first reported biochemical inhibitor of FTO, acts as a competitive binder to the active site of FTO [193]. However, there is evidence suggesting that rhein not only inhibits FTO activity but also affects ALKBH2, indicating that it is not a specific inhibitor of FTO [72]. Meclofenamic acid (MA), an anti‐inflammatory drug, has been identified as a selective inhibitor of FTO activity, competing with the binding site of FTO and suppressing its activity [194]. R‐2‐hydroxyglutarate also demonstrates the ability to suppress FTO activity while serving an anti‐cancer role [195]. Furthermore, AI‐based approaches have been employed to develop a variety of FTO inhibitors, identifying diacerein (IC50 = 1.51 μM) and entacapone (IC50 = 3.5 μM) as potent FTO inhibitors [196, 197], with diacerein shown to bind directly to FTO.

In conclusion, METTL3 and FTO represent promising targets for drug development in the treatment of disease. The development of inhibitors or activators of other m^6^A methylases is warranted, and dedicated inhibitors for osteoporosis treatment should be further explored. Exosomes extracted from the serum of PMOP patients can be utilised to investigate the role of m6A regulators. Seven diagnosable m6A regulators have been identified, including FTO, FMR1, YTHDC2, HNRNPC, RBM15, RBM15B and WTAP [198]. A study involving 80 women with varying BMD analysed differences in the expression levels of the major m6A regulators, leading to the identification of four potential biomarkers for osteoporosis diagnosis: METTL16, CBLL1, YTHDF2 and FTO [199]. Additionally, other research has highlighted FTO, YTHDF2 and CBLL1 as diagnostic biomarkers and m6A‐related molecular patterns in osteoporosis [200].

## 5 Conclusion

Recent research has elucidated the pivotal roles of m^6^A in the pathogenesis of various diseases [36]. The abnormal expression of m^6^A, governed by distinct regulators, affects cellular functions and fates in conditions such as cancer [201], multiple sclerosis [202], rheumatoid arthritis (RA) [203, 204], ischaemic stroke (IS) [205, 206] and obesity [207, 208]. Furthermore, this paper emphasises that the current understanding of m^6^A in osteoporosis remains limited. Most studies have primarily concentrated on the ‘writers’ and ‘erasers’ of m^6^A, with relatively few reports addressing the role of ‘reader’, despite the investigation of ‘readers’ in other diseases. For instance, IGF2BP1 enhances mRNA stability and has been demonstrated to combine with circPTPRA in the cytoplasm of bladder cancer cells [209]; additionally, the upregulation of LRPPRC is closely related to prognosis, survival and resistance in prostate cancer [210]. Moreover, the function of bone resorption mediated by osteoclasts is a crucial aspect of the bone remodelling process and plays an important role in maintaining bone structure. m^6^A also regulates the proliferation and differentiation of osteoclasts involving METTL3, METTL14, YTHDF2 and ALKBH5; however, further correlational studies are needed to elucidate the roles of other methylases. Finally, current drugs targeting m^6^A mainly focus on METTL3 and FTO, with research predominantly centred on cancer. There remains a lack of drugs specifically targeting osteoporosis, and further investigation into their effectiveness and safety is necessary.

Numerous studies have explored the potential of natural products from traditional medicine in targeting m^6^A modifications in cancer. For instance, the combination of resveratrol and curcumin, both phenolic compounds, has been shown to enhance the growth and integrity of the intestinal mucosa by enhancing the expression of YTHDF2 in the ileum [41]. Similarly, epigallocatechin gallate, a flavonoid with anti‐inflammatory, antioxidant and anticancer properties, can modulate the cyclin CDK2 and A2 by reducing the levels of FTO and raising the expression of the ‘readers’ YTHDF2 through m^6^A mediated patyways [211]. Research on betaine, a natural alkaloid, has demonstrated its inhibitory effect on the expression of the ‘writers’ METTL3 and METTL14 in HepG2 cells while also having a helpful effect on the expression of the ‘erasers’ FTO and ALKBH5 [212]. Furthermore, compounds such as claudine E and camptothecin have been found to directly inhibit the bioactivity of FTO [45, 46]. Other natural products and their derivatives, including saikosaponin D [213], fusaric acid [214], sulforaphane [215] and quercetin [216], have also been reported to exert anticancer activity through the regulation of m^6^A pathways. Natural products and their derivatives offer valuable opportunities for the treatment of osteoporosis, as evidenced by numerous reports. For instance, icariin has been shown to upregulate METTL14‐mediated m^6^A modification of the prolyl 4‐hydroxylase beta subunit (P4Hβ), thereby promoting the osteogenic differentiation of BMSCs [134]. Additionally, the extract of *Eclipta prostrata* (L.) L. and its component wedelolactone enhance the osteoblastogenesis of BMSCs by targeting METTL3‐mediated m6A RNA methylation [126]. Furthermore, there are reports highlighting the promotion of osteogenic differentiation through m^6^A modification in Chinese patent medicines, including the Xianling Gubao Capsule [217] and Qianggu Decoction [218].

This paper explores the mechanism of m^6^A modification in relation to bone remodelling in BMSCs, osteoblasts and osteoclasts. It also reviews the effects of key osteogenic transcription factors, signalling pathways related to bone homeostasis, nc RNAs, autophagy and other factors on bone metabolism through m^6^A modification, as well as the development of related inhibitors and agonists that were reviewed. Currently, research on the role of m^6^A modification in osteoporosis primarily relies on animal studies, with relatively few clinical investigations conducted. Consequently, the experimental methods and clinical detection techniques associated with m^6^A require further exploration.

## Author Contributions

**Shuo Tian:** conceptualization (equal), funding acquisition (equal), writing – original draft (equal). **Yagang Song:** conceptualization (equal), investigation (equal), writing – original draft (equal). **Lin Guo:** data curation (equal), visualization (equal), writing – review and editing (equal). **Hui Zhao:** formal analysis (equal), writing – review and editing (equal). **Ming Bai:** project administration (equal), supervision (equal), writing – review and editing (equal). **Mingsan Miao:** conceptualization (equal), funding acquisition (equal), project administration (equal), supervision (equal), writing – review and editing (equal).

## Conflicts of Interest

The authors declare no conflicts of interest.

## Data Availability

The authors have nothing to report.
